# Ontogenetic shifts in sound production and shared sonic mechanisms in two priacanthid fishes

**DOI:** 10.7717/peerj.20821

**Published:** 2026-02-26

**Authors:** Marine Banse, Alexy Pécret, David Lecchini, Eric Parmentier

**Affiliations:** 1 Laboratoire de Morphologie Fonctionnelle et Evolutive, Université de Liège, Liège, Belgium; 2 PSL University, EPHE-UPVD-CNRS, USR 3278 CRIOBE, Moorea, French Polynesia; 3 Laboratoire d’Excellence “CORAIL”, Perpignan, France

**Keywords:** Acoustic communication, Sound production, Sound producing-mechanism, Priacanthidae, Sonic muscles

## Abstract

Sound production in teleost fishes relies on diverse anatomical adaptations, yet convergent mechanisms involving extrinsic sonic muscles acting on the swim bladder are widespread. This study investigates the acoustic and morphological features of two priacanthid species, Indo-Pacific glasseye *Heteropriacanthus carolinus* and moontail bullseye *Priacanthus hamrur* to explore interspecific similarities in sound production. Using recordings and anatomical analyses, we show that both species rely on a forced-response mechanism, where the contraction rate of fast sonic muscles determines the fundamental frequency. This is corroborated by the smaller diameter of sonic fibres compared to epaxial fibres in both species. Despite belonging to different genera, both species exhibit extrinsic sonic muscles originating from the first pleural rib and inserting on the anterior swim bladder. However, *P. hamrur* displays anterior bladder projections potentially involved in enhanced hearing, absent in *H. carolinus*. Acoustic signals were broadly similar between species, suggesting that the morphological shift in muscle insertion does not affect sound structure. Comparative analysis across geographically distant populations of *H. carolinus* (Indian and Pacific Oceans) revealed variation in acoustic features that was size-dependent rather than region-specific. Juveniles emitted continuous pulse trains with high fundamental frequencies, whereas adults produced more segmented calls with lower frequencies. These ontogenetic differences reflect developmental modulation of vocal output, not anatomical changes. Overall, our findings highlight the conserved nature of sonic mechanisms in Priacanthidae, the influence of body size on acoustic variation, and the potential role of swim bladder morphology in auditory enhancement rather than sound generation.

## Introduction

In teleosts, numerous sound-producing mechanisms have evolved independently across different lineages (Fine & Parmentier, 2015; Looby et al., 2022), resulting in a remarkable diversity of sonic systems. One of the most widespread mechanisms, arising through evolutionary convergence (Parmentier & Fine, 2016), involves fast-contracting muscles that act upon the swim bladder. Depending on the taxon, sonic muscles exhibit various types of anatomical insertions. In the most general terms, these muscles can be classified as either intrinsic or extrinsic relative to the swim bladder. Intrinsic sonic muscles originate and insert directly on the swim bladder itself, as observed in several Batrachoididae (Barimo & Fine, 1998; Rice & Bass, 2009), Zeidae (Onuki & Somiya, 2004), some Ostraciidae (Parmentier et al., 2020) and Triglidae (Connaughton, 2004). Extrinsic sonic muscles, by contrast, have only one point of attachment to the swim bladder. This insertion may be direct, as in Pempheridae (Takayama et al., 2003; Parmentier, Fine & Mok, 2016) or Gadidae (Brawn, 1961), or may involve an intermediary structure closely associated with the swim bladder. For instance, in numerous catfish such as those in the Mochokidae (Kaatz & Stewart, 2012; Boyle, Colleye & Parmentier, 2014), Malapteruridae (Boyle, Bolen & Parmentier, 2015) and Doradidae (Kaatz & Lobel, 2001; Boyle et al., 2015) families, the sonic muscle inserts onto a bony structure called the Müllerian ramus (Muller, 1842), located anterior to the swim bladder. The use of ribs intimately associated with the swim bladder as insertion points for sonic muscles is a feature still observed in many Holocentridae species (Parmentier et al., 2011; Banse et al., 2024). In other taxa, the muscle may form a sheet-like structure (nappe) that encircles the swim bladder either ventrally, as seen in piranhas (Nelson, 1961; Smith, 1992; Mélotte et al., 2019; Banse et al., 2021), or dorsally, as in Sciaenidae (Vance et al., 2002; Lagardère & Mariani, 2006; Parmentier et al., 2014). In these cases, the opposing end of the muscle typically inserts on adjacent skeletal elements such as the skull, pectoral girdle, or vertebrae.

Priacanthids, commonly called the bigeyes, are a relatively small circumtropical family of fishes consisting of 21 species within four genera. They live in epibenthic habitats and are generally associated with rock formations or coral reefs where they shelter in and near crevices and beneath ledges for most of the daytime hours and possibly at night (Fernandez-Silva & Ho, 2017). However, trawling data indicate that a few species can occur in more open bottom areas. In Priacanthidae, little information about sound communication exists. Some sounds have been reported for *Heteropriacanthus cruentatus*, *Priacanthus macracanthus* and *Priacanthus meeki* (Salmon & Winn, 1966) but an accurate description of the mechanism and of the sounds they produce is still lacking. In *H. cruentatus*, Salmon & Winn (1966) described that most of the sounds lasted between 300 and 600 ms and have a dominant frequency that ranges from 150 to 300 Hz. In this species, sounds could be produced by sonic muscles acting on the swim bladder (Walls, 1964; Salmon & Winn, 1966). These muscles originate on the first rib and insert on the anteroventral part of the swim bladder (Starnes, 1988). Although first considered to be circumtropical, studies have recently suggested that the previously recognized monogenic *H. cruentatus* is composed of three different species (Gaither et al., 2015; Fernandez-Silva & Ho, 2017). The first species, *H. cruentatus*, occurs in the western Atlantic, from Mexico to Brazil. The second one, *H. fulgens* is found in the north-eastern Atlantic, in the Canary Islands and Cape Verde. Finally, *H. carolinus* lives in the Indian and Pacific Oceans, from Madagascar and the Red Sea to Hawaii and French Polynesia. *Heteropriacanthus carolinus* (Cuvier, 1829) is a 30 cm long, red and large eyed fish, mainly living on coral reefs, between 5 and 200 m depth, solitary during the day, but forming groups during night and dusk to feed (Salmon & Winn, 1966).

Previous studies on various fish species have demonstrated linear relationships between certain acoustic features and individual size (Ladich, 2004). These relationships can generally be categorized into two groups, based on the underlying sound production mechanisms (Parmentier & Fine, 2016). In both cases, larger individuals tend to produce sounds with lower dominant frequencies. However, in species where sound production relies on a forced-response mechanism, where the frequency is directly linked to the contraction rate of high-speed sonic muscles (*e.g.*, Sciaenidae, Batrachoididae, Serrasalmidae), the slope of the frequency-size relationship does not exceed 5% (Skoglund, 1961; Fine, 1978; Fine & Waybright, 2015). This implies that the emitted sound does not convey reliable information about the size of the sender. In contrast, species whose sound production does not involve high-speed muscles (*e.g.*, Pomacentridae, Cichlidae) exhibit a much steeper slope, often exceeding 60% (Myrberg Jr, Ha & Shamblott, 1993; Malavasi et al., 2003; Colleye et al., 2011; Bertucci et al., 2012), suggesting that the acoustic signal can serve as an indicator of the sender size (Parmentier & Fine, 2016). Notably, this linear relationship is not observed in the triggerfish *Rhinecanthus aculeatus*. In this species, dominant frequency decreases exponentially with body size in juveniles, before reaching a plateau between 60 and 85 mm total length (TL), beyond which larger individuals (up to 175 mm TL) emit sounds with a constant dominant frequency (Parmentier et al., 2017).

The objectives of this study were: (1) First, to describe and compare the calls and the sound-producing mechanisms of *H. carolinus* and *Priacanthus hamrur,* the only two soniferous genera within Priacanthidae, in order to determine whether differences in anatomy can be reflected in their sounds; (2) to compare sounds of *H. carolinus* from the Indian and Pacific Oceans to assess whether they differ between populations. This secondary aim of the study nevertheless yielded unexpected insights, namely that juveniles and adults of *H. carolinus* can exhibit substantial differences in the acoustic parameters characterizing their sounds.

## Materials & Methods

### Data collection

**Figure 1:**
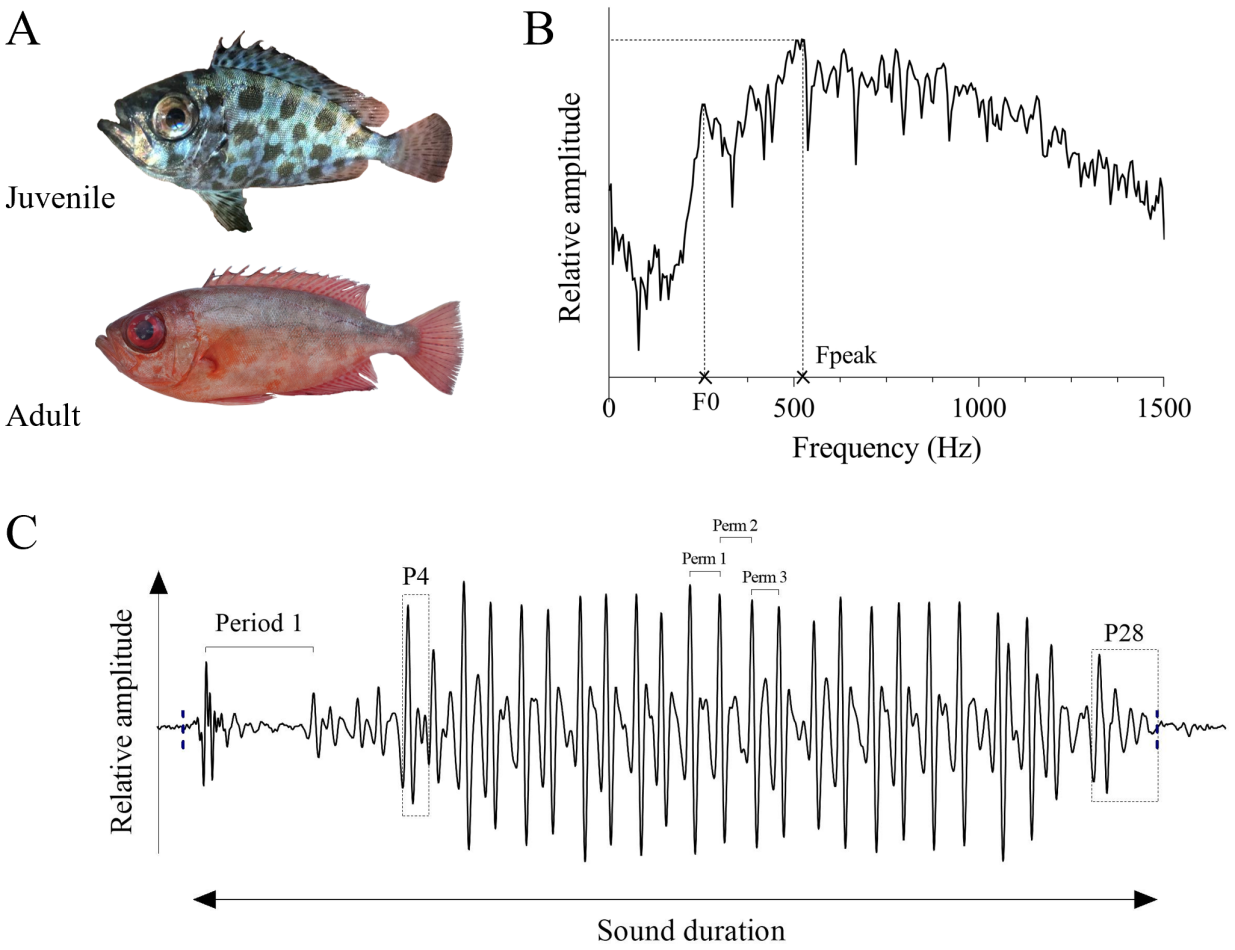
Illustration of *Heteropriacanthus carolinus* individuals and their calls. (A) Photographs of a juvenile (TL: 7.8 cm) and an adult (TL: 25.8 cm); (C) oscillogram of a representative sound of a juvenile with corresponding (B) power spectra. P pulses, Perm 1-3 pulse periods from the middle of each sound, F_1_ fundamental frequency, Fpeak dominant frequency.

Eleven specimens of *H. carolinus* belonging to three populations (7.8–28 cm total length (TL)) were collected using hand nets, between August 2020 and April 2022, by snorkeling at night in coral reef areas of French Polynesia, Guam and Seychelles at a depth between 2 and 20 m (Table 1). Specimens were placed in a floating fish basket and recorded directly after their capture. Three of specimens of the Seychelles were, following recordings, euthanized in a tricaine methanesulfonate solution (MS-222) for subsequent morphological investigations. All other individuals were released. Based on the length at first maturity (between 18 and 20 cm) reported in the literature in a few priacanthid species (Saker et al., 2015; Jabbar et al., 2018), individuals belonged to distinct developmental classes: one group consisting of juveniles and the other of adults (Fig. 1A). Additionally, we purchased three specimens of *P. hamrur* (16.5–21.5 cm TL) from the aquarium trade (Table 1) and housed them in the Laboratory of Functional and Evolutionary Morphology (Liège University, Belgium). Fish were maintained in a 200-l saltwater tank at 26 °C (12 h:12 h light:dark cycle) with artificial plants and clay pots and fed three times a week with mussels. These three specimens were, as with those of *H. carolinus,* euthanized in a MS-222 solution following the recordings, for subsequent morphological investigations. All procedures were approved by the ethical commission of Liège University (protocol 1759).

**Table 1:** Summary of mean ± sd, calculated from n sounds for each individual, for the different acoustical variables.

Species	Origin	ID	TL	*n*	Sound duration (ms)	Number of pulses	F0 (Hz)	Dominant frequency (Hz)	Period 1 (ms)	Middle period mean (ms)
*H. carolinus*	Guam	1	7.8	20	231.6 ± 109.9	56 ± 27.5	255 ± 9	570 ± 90	6.1 ± 3.6	4 ± 0.2
	PF	1	8.1	20	128 ± 77	29.8 ± 17.6	258 ± 17	525 ± 36	8.3 ± 6	3.9 ± 0.3
	PF	2	7.8	20	184 ± 90.6	43.6 ± 21.9	251 ± 8	551 ± 77	4.7 ± 3.4	4.2 ± 0.3
	PF	3	7.8	20	331.6 ± 191.5	75 ± 44.4	246 ± 15	772 ± 98	4.9 ± 2.7	4.4 ± 0.4
	PF	4	11	20	418.3 ± 139.1	87 ± 30.9	237 ± 10	560 ± 69	13.9 ± 3.6	4.4 ± 0.4
	PF	5	8.1	20	154.1 ± 91.2	37.1 ± 23.9	254 ± 7	695 ± 79	7.2 ± 2.1	4 ± 0.3
	SEY	1	8.4	20	128.5 ± 59.5	28.4 ± 13.5	255 ± 13	582 ± 68	7.9 ± 4.7	4 ± 0.6
		**JUVENILE MEANS**			140	225 ± 111	51 ± 23	251 ± 7	608 ± 90	8 ± 3	4 ± 0
	SEY	2^*^	25.8	20	177 ± 72.5	10.1 ± 3.3	60 ± 15	364 ± 44	15.8 ± 1.7	17.9 ± 2.1
	SEY	3^*^	25.5	20	251.6 ± 115.2	12.3 ± 5.1	48 ± 3	343 ± 47	20.5 ± 3	21.2 ± 3.1
	SEY	4^*^	28	11	290.8 ± 179.3	18.6 ± 9.4	80 ± 35	523 ± 185	18.6 ± 8.4	14.8 ± 5.4
	SEY	5^*^	26.3	13	157.3 ± 67.9	7.2 ± 2.7	48 ± 3	397 ± 58	20.5 ± 2.5	22.7 ± 2.8
	**ADULT MEANS**			64	219 ± 63	12 ± 5	59 ± 15	407 ± 81	19 ± 2	19 ± 4
*P. hamrur*	AT	1^*^	16.5	20	87.3 ± 12.4	11.7 ± 1.6	136 ± 3	251 ± 50	6.8 ± 0.4	7.4 ± 0.2
	AT	2^*^	21.5	20	96.5 ± 7.5	12.6 ± 1	139 ± 2	268 ± 30	6.1 ± 0.8	7.3 ± 0.1
	AT	3^*^	18.5	20	153.7 ± 76.4	19 ± 10.1	129 ± 21	276 ± 80	8.2 ± 3.1	8.3 ± 2.4
	**ADULT MEANS**			60	112 ± 36	14 ± 4	135 ± 5	265 ± 13	7 ± 1	8 ± 1

**Notes.**

AT aquarium trade F1 fundamental frequency PF French Polynesia SEY Seychelles TL total length (cm)

* Adult specimens.

Sounds produced by fish were first recorded, before being measured in total length. As sexual dimorphism has never been reported in this fish family, the sex of the individuals was unknown.

### Sound recording

The sounds of specimens of *H. carolinus* were recorded at sea to avoid sound distorsion (Banse et al., 2023). Water temperature during recordings ranged between 28 °C and 30 °C. Since *P. hamrur* were acquired in the laboratory, the sounds were necessarily recorded in a tank, specifically a second 200-L saltwater tank maintained at 26 °C. Recordings were conducted using a High Tech Inc. HTI-96-MIN hydrophone (sensitivity: −164.4 dB re 1 V/µPa; Long Beach, CA, USA) connected to a TASCAM DR-05X recorder (Milton Keynes, UK). During the recordings, fish were manually held 15 cm below the surface and positioned approximately five cm from the hydrophone, with their mouths facing the sensor. This distance also enables fish to remain within the attenuation distance (Akamatsu et al., 2002). Moreover, recordings in small tanks induce potential artifacts because of reflections and tank resonance, and an estimated minimum resonant frequencies of 2,250 Hz was calculated following the equation of Akamatsu et al. (2002). A low-pass filter (two kHz) was consequently applied to all recordings. All tested fish produced sounds under this protocol, with approximately 60 sounds recorded per individual. For analysis, the 20 highest-quality sounds (those with the best signal-to-noise ratios) were selected. In a few instances where individuals were less cooperative, fewer recordings were obtained. This paper does not concern fish behavior.

### Sound analysis

Sounds were manually analyzed using Avisoft-SAS Lab Pro 5.2.13 (Avisoft Bioacoustics, Glienicke, Germany). Recordings were digitized at a sampling rate of 44.1 kHz with 16-bit resolution and subsequently band-pass filtered between 50 and 2,000 Hz. Six standard acoustic parameters were then measured from the sounds (Figs. 1B, 1C): (1) sound duration (milliseconds, ms), (2) number of pulses per sound, (3) first pulse period (defined as the peak-to-peak interval between the first two pulses, ms), (4) three pulse periods from the middle of each sound (Perm 1, 2 and 3, measured on oscillograms as the peak-to-peak time duration between two consecutive pulses, ms), and as previously described in Banse et al. (2024), (5) fundamental frequency (F_1_, defined as the lowest frequency of a harmonic sound, Hz) and (6) dominant frequency (defined as the frequency with the greatest energy, Hz) that were extracted from the power spectrum of the whole sound (Hamming window).

### Anatomy

Euthanized specimens were directly placed in a 5% formalin solution for 2 days for fixation and then transferred to 70% ethyl alcohol.

#### 3D reconstruction of the sound-producing apparatuses

Investigations of the sound-producing mechanisms were first based on 3D reconstructions from µCT scans in *H. carolinus*. Micro CT scanning of *H. carolinus* specimen was completed using a FleXCT system consisting of a customized UniTOMXL scanner from Tescan XRE (Samber De et al., 2021). Images of *H. carolinus* were acquired at 100 kV and 1,000 µA, resulting in 1,440 images with a voxel size of 30 µm. Image reconstruction was carried out using Panthera (Tescan XRE). Segmentation, visualization, and analysis were performed in Aquila TM software while three-dimensional (3D) datasets were exported in 16-bit format and later converted to 8-bit voxels using ImageJ (Abramoff, Magalhaes & Ram, 2014). Direct volume renderings (iso-surface reconstructions) were then used in AMIRA 2019.2 to visualize selected voxel subsets corresponding to the skeleton, muscles, and swim bladder. Following µCT scans, three specimens of each species were dissected and examined using a Wild M10 binocular microscope (Leica Microsystems GmbH, Germany) equipped with a camera lucida to be able to describe the structures involved in the sound-producing mechanism. These data were used to complete the descriptions of the skeleton and swim bladder. The images of the whole specimen and the 3D models of the sound-producing apparatus are available on the online repository MorphoSource (https://www.morphosource.org/concern/media/000765108; https://www.morphosource.org/concern/media/000765146).

#### Histological analysis

After dissection, the left muscle associated with the swim bladder and the white epaxial fibers from both species were collected. Each sample was dehydrated in butanol, decalcified, embedded in paraffin, and serially sectioned at 10 µm using a Reichert microtome. Sections were then stained with hematoxylin and examined under a VHX-7000 digital microscope (Keyence, Osaka, Japan), which enabled measurements on the cross-sections.

### Statistical analysis

Descriptive statistics were calculated for each temporal and spectral property of the acoustic signals produced by each individual.

We first investigated sound variation among the populations of *H. carolinus* of the Pacific and Indian Oceans. Since recorded individuals of *H. carolinus* comprised both juveniles and adults and that previous studies on various species have demonstrated linear relationships between certain acoustic features and fish size (Ladich, 2004), regressions were used to assess the relationships between total length (TL) and the acoustical parameters. For each acoustic variable, we first tested whether it was significantly correlated with TL using linear regressions. Variables showing significant correlations with TL were then divided by TL to correct for size effects, following approaches commonly used in fish bioacoustics (Raick et al., 2020; Horvatić et al., 2021; Banse et al., 2024). To determine if sounds could be discriminated between the populations of the two oceans, we carried out a principal component analysis (PCA). For the interpretation of PCA results, we considered the number of factors equivalent to the number of eigenvalues greater than 1.

A direct statistical comparison between *P. hamrur* and *H. carolinus* was not performed, as such a test would have limited interpretative value. With only two species considered, interspecific differences could not be meaningfully generalized. A broader comparative framework involving multiple species across Priacanthidae would be required to draw evolutionary or functional inferences from statistical contrasts.

The morphological features were first tested for the assumption of normality (Shapiro–Wilk test) and homoscedasticity of variances (Levene’s test). Mann–Whitney-Wilcoxon tests were then used to compare the diameter of sonic and epaxial muscle fibres in *H. carolinus .* Statistical analyses were carried out with RStudio version 2025.05.0 (RStudio Team, 2025) and GraphPad version 5 (GraphPad Software, Inc., La Jolla, CA, USA).

## Results

### Sounds

#### Sounds description

**Figure 2:**
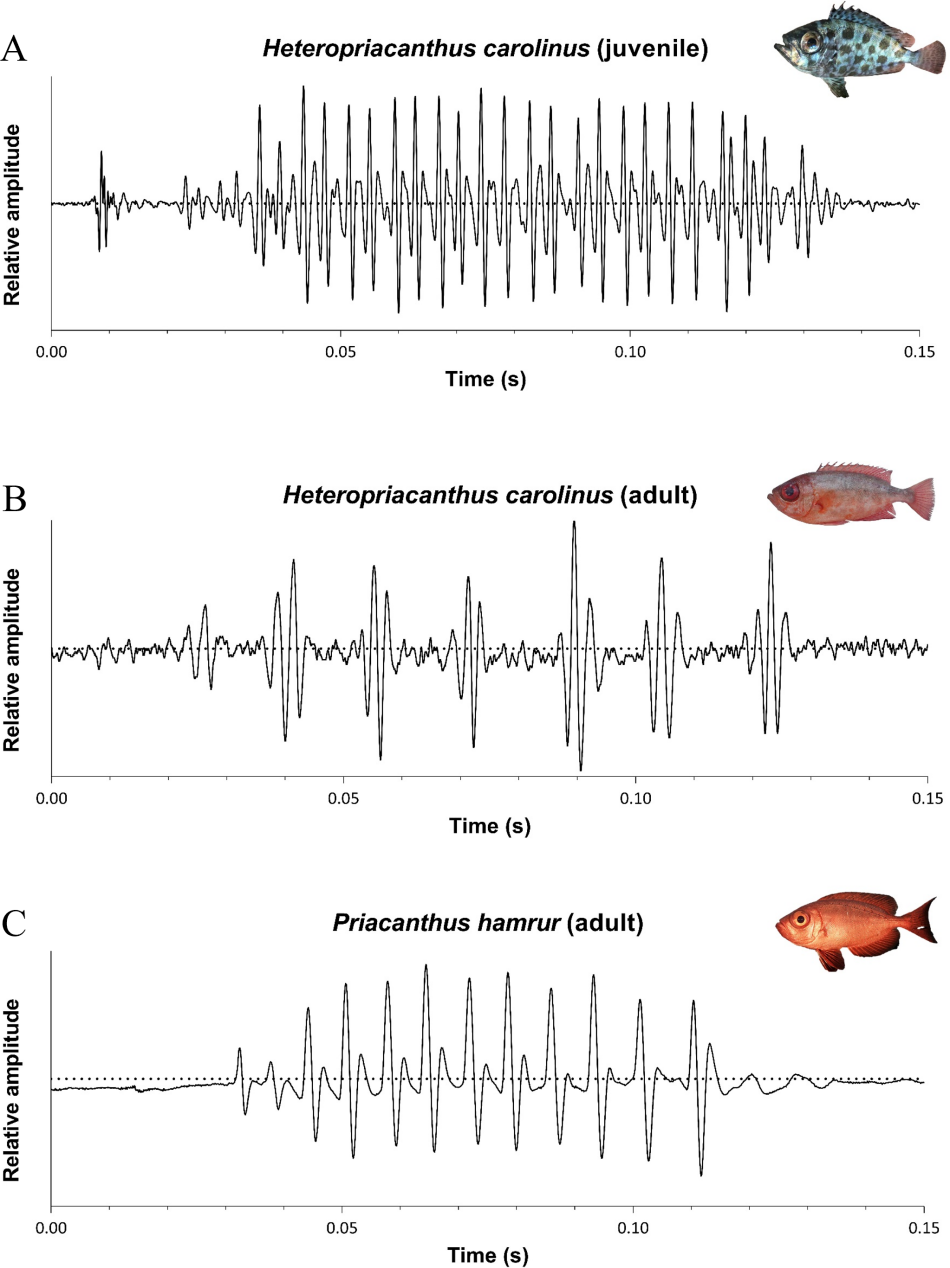
Oscillograms of the sounds produced by (A) juvenile and (B) adult specimens of *Heteropriacanthus carolinus* and (C) *Priacanthus hamrur*. Photograph of *P. hamrur*: Randall, J.E., *via* FishBase.

*Heteropriacanthus carolinus* produces sounds lasting between 128 and 418 ms (Fig. 2; Table 1). The dominant frequency varies between 343 and 772 Hz. Sounds of adult individuals are generally composed of seven to 19 pulses, have a fundamental frequency ranging from 48 to 80 Hz and their periods between 15 and 23 ms (Fig. 2B). Sounds produced by juveniles are composed of 28 to 87 pulses. Their fundamental frequency is about 250 Hz and their periods vary between four and 14 ms (Fig. 2A).

*Priacanthus hamrur* produces sounds lasting between 87 and 154 ms and composed of 12 to 19 pulses, with periods of 6 to 8 ms (Fig. 2C; Table 1). Their fundamental frequency is about 135 Hz whereas their dominant frequency is about 260 Hz.

**Figure 3:**
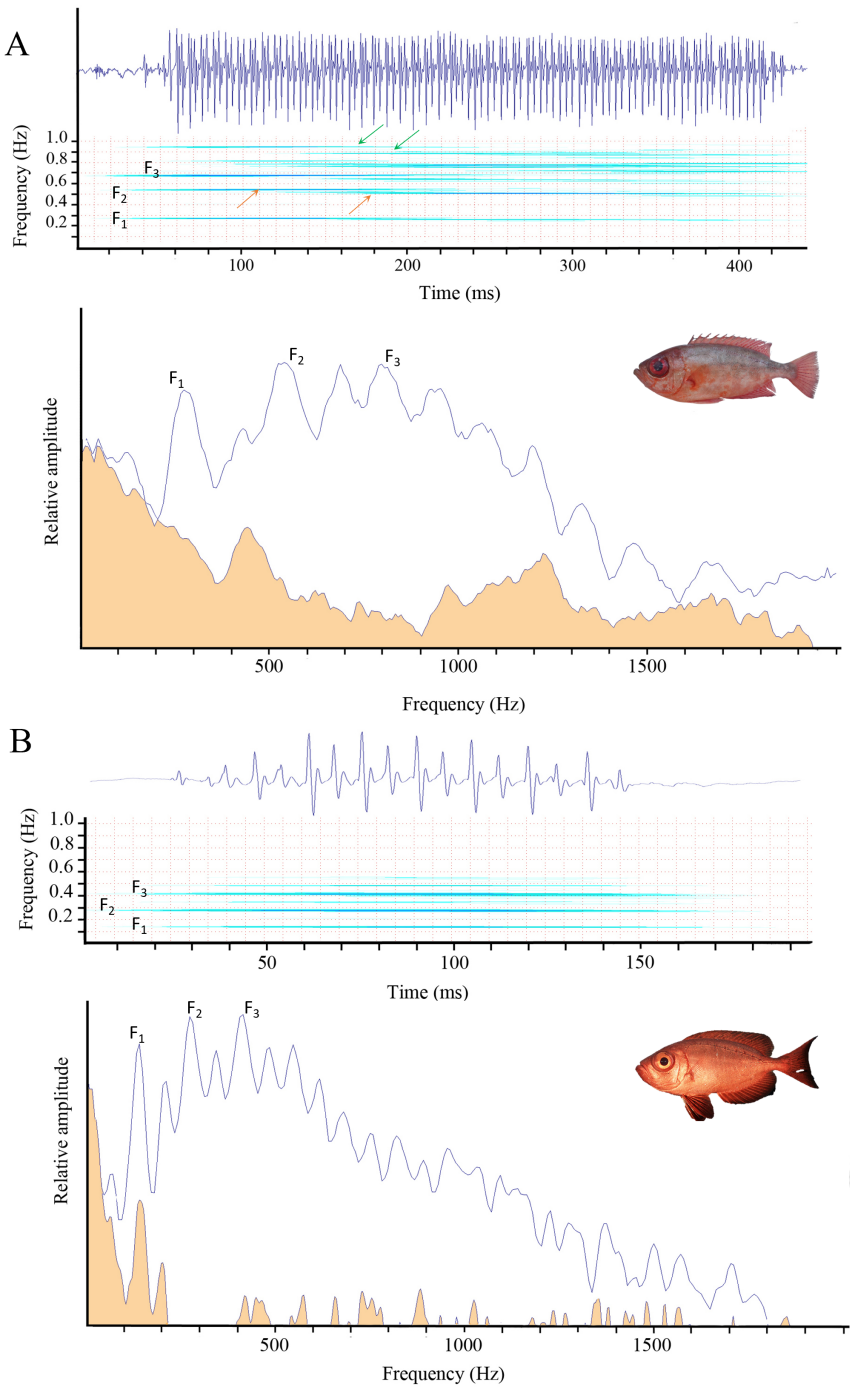
From top to bottom: oscillogram, spectrogram, and power spectrum of sounds in *Heteropriacanthus carolinus* (A) and *Priacanthus hamrur* (B). F_1_ corresponds to the fundamental frequency, and F_2_ and F_3_ to the first harmonics. The orange shading indicates background noise. Arrows highlight frequency modulations resulting from variations in the pulse period. Photograph of *P. hamrur*: Randall, J.E., *via* FishBase.

In both species, variations in pulse period result in slight modulations of the fundamental frequency (F_1_), which may also affect the harmonic structure. Thus, F_1_ is not strictly constant within a call (Fig. 3).

#### Relationship between size and acoustical variables

In *H. carolinus*, significant relationships were found between TL and all acoustical variables, except the sound duration (*p* < 0.05; Table S1). These variables correlated with size were divided by total body length for subsequent analyses, following the formula “*X* (TL)^−1^” where “*X*” is the acoustical variable. This allowed appropriate comparison by reducing the effect of fish size on acoustic variability (Raick et al., 2020; Horvatić et al., 2021; Banse et al., 2024). Acoustic variables of sounds produced by *P. hamrur* were, similarly to *H. carolinus* (all except sound duration), divided by total body length.

#### Sound comparison between the Indian and the Pacific Oceans

**Figure 4:**
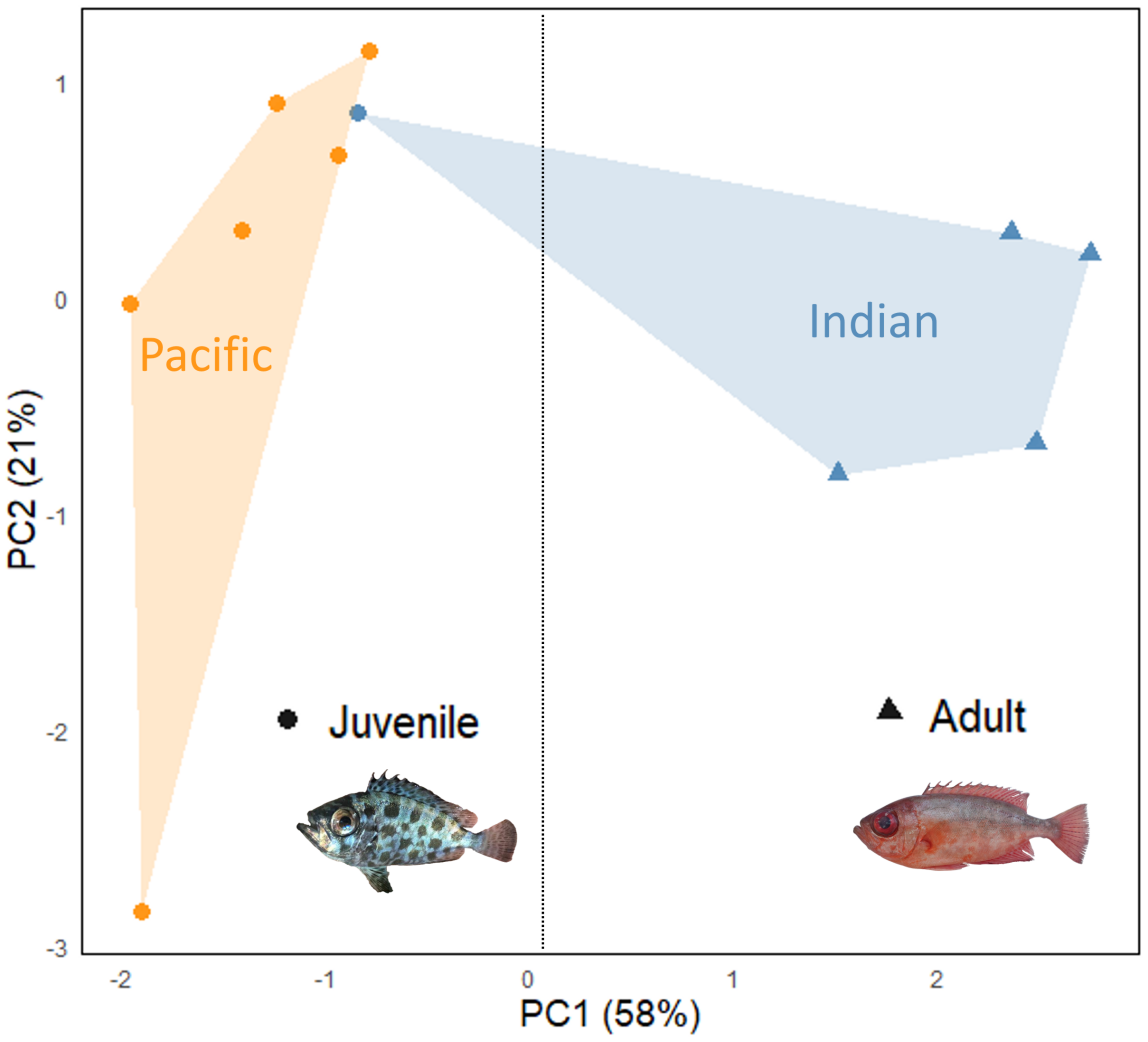
Scatterplot of the first two principal components (PC1 and PC2) based on acoustic variables of sounds in populations of *H. carolinus* from the Indian and Pacific Oceans. The grouping by population, however, appears to be a decoy, since individuals are rather gathered by size (juvenile *vs* adult).

PC1 and PC2 explained 58 and 21% of the acoustical variation, respectively, for a total explained variation of 79%. The number of pulses in sounds, the fundamental and dominant frequencies and the middle pulse period mean mostly contributed to PC1, whereas the sound duration and the first period mainly contributed to PC2 (Table S2). Although individuals cluster by ocean, the left and right parts of the PCA actually group juveniles and adult specimens together, respectively. Despite the correction made to reduce the size effect on the acoustic variables, there is an effect related to the developmental stage. Indeed, the sole individual from the Seychelles found on the left part of the PCA corresponds to the only juvenile recorded in this population, and only juveniles were recorded in Guam and French Polynesia, whereas the four adult individuals recorded in the Seychelles occupy the right part of the PCA. Thus the acoustic differences observed among *H. carolinus* populations appear to be primarily size-related (Table 1, Fig. 4). Notably, the only juvenile from the Indian Ocean with a body size comparable to that of Pacific individuals exhibited acoustic features closely resembling those of Pacific juveniles. Therefore, the observed differences in sound characteristics primarily reflect a developmental stage rather than geographic variation. This ontogenetic pattern is clearly visible in the oscillograms: juveniles produce continuous pulse trains, whereas in adults, pulses are spaced apart (Fig. 3).

### Anatomy

#### Morphology of the sound-producing apparatus

##### Heteropriacanthus carolinus.

**Figure 5:**
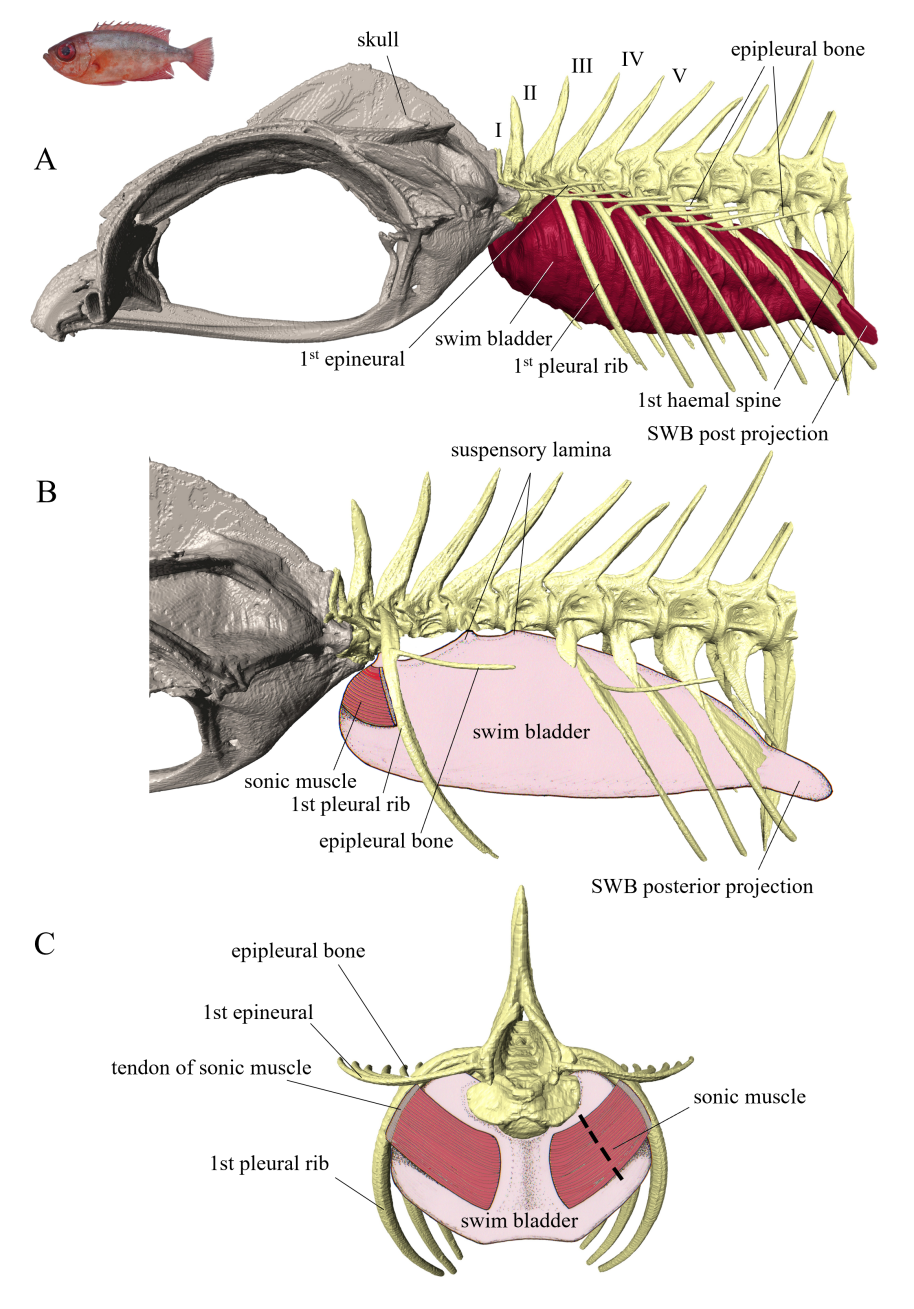
Left lateral (A, B) and frontal (C) views of the sound-producing apparatus in *Heteropriacanthus carolinus*. (A) view showing the general organization of the swim bladder and its relationship to the vertebral axis. In (B) several epipleural and pleural bones have been removed to reveal the suspensory lamina and the arrangement of the sound-producing muscle. Dotted line corresponds to histological cross section (see Fig. 7).

**Figure 6:**
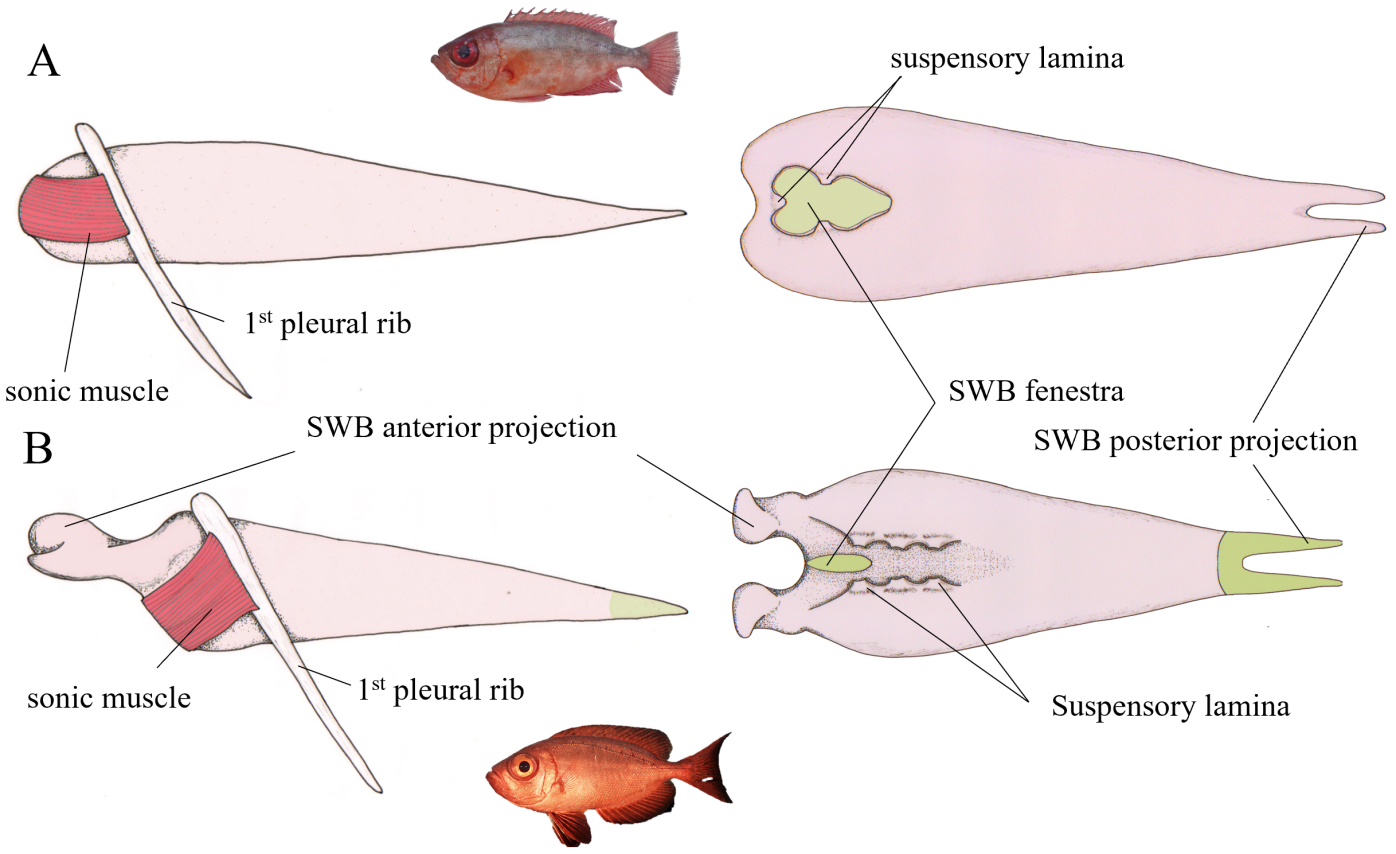
Left lateral and dorsal schematic view of the swim bladder in *Heteropriacanthus carolinus* (A) and *Priacanthus hamrur* (B). Green areas are parts of the swim bladder deprived of tunica externa. Suspensory laminae are thin connective laminae arising from the swim bladder wall (tunica externa), anchoring it to the vertebral column. The anterior projections of the swim bladder in *P. hamrur* are positioned against the posterior part of the otic cavity of the skull. The anterior projection of the swim bladder seems to be related with the ventral displacement of the sound-producing muscle. Photograph of *P. hamrur*: Randall, J.E., *via* FishBase.

The oblong swim bladder forms caudally two projections (Y-shaped) that surround the pterygiophores of the anal fin (Figs. 5A–5C). The tunica externa exhibits three points of attachment: the first is located between vertebrae I and II, while the second and third correspond to narrow lateral extensions of the swim bladder that connect to the flanks of vertebrae III and IV. Dorsally, between these points of attachment to the vertebral column, a region lacking tunica externa exposes the underlying tunica interna, forming a swim bladder fenestra (Fig. 6A). Extrinsic sonic muscles have a tendinous origin on the anterior margin of the first pleural rib and insert on the anterior part of the swim bladder. This arrangement results in a muscle fibre orientation that is perpendicular to the rib and parallel to the fish axis. Contractions of these sonic muscles likely compress the anterior region of the swim bladder, thereby displacing internal gas toward the posterior chamber and caudal projections. This displacement may generate vibrations that are transmitted through the swim bladder fenestra, contributing to sound radiation.

##### Priacanthus hamrur.

The swim bladder possesses not only posterior projections but also two anterior projections that are associated with the saccular fossae of the skull (Fig. 6). At the level of the posterior projection, the swim bladder lacks a tunica externa. It exhibits a small dorsal swim bladder fenestra at the anterior end and the swim bladder is closely associated dorsally with the vertebrae of the spinal column. Extrinsic swim bladder muscles are present (Fig. 6); they originate on the first pleural rib and insert on the anterior portion of the swim bladder, just behind the origin of the anterior projections.

#### Histology of the sonic muscles

**Figure 7:**
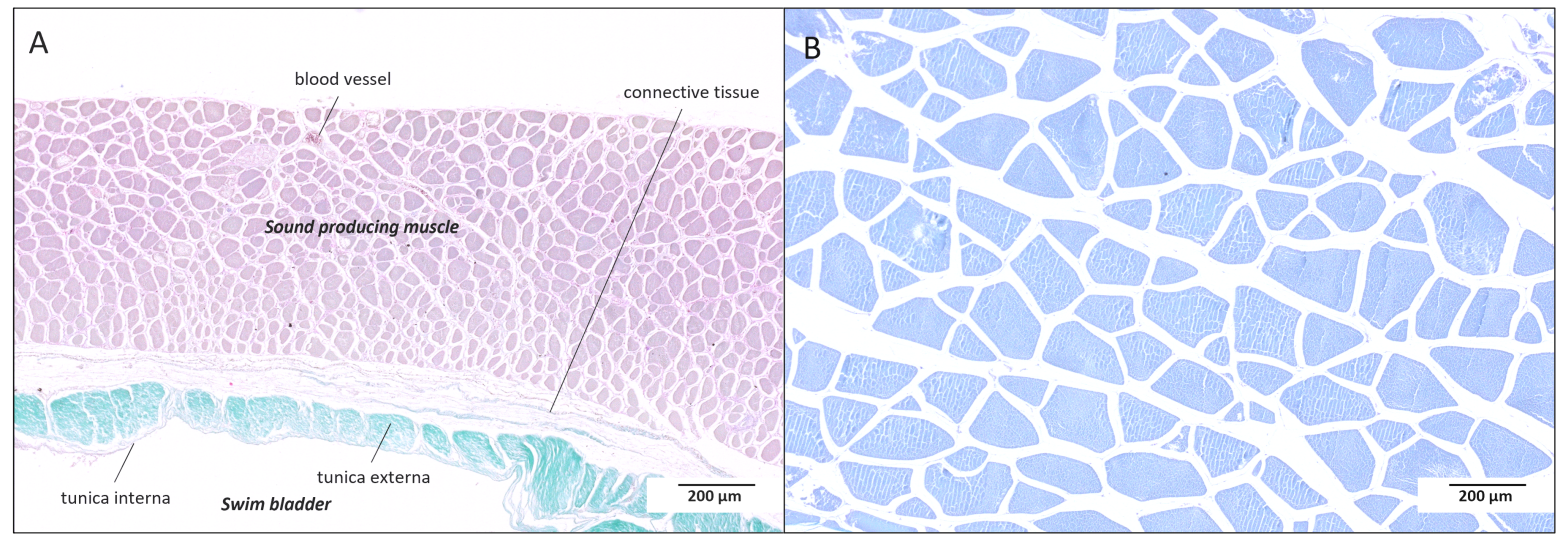
Cross sections in the sound-producing muscle (A) and in the white epaxial muscles (B) in *Heteropriacanthus carolinus* to show dramatic differences in the area of the muscle cells.

**Figure 8:**
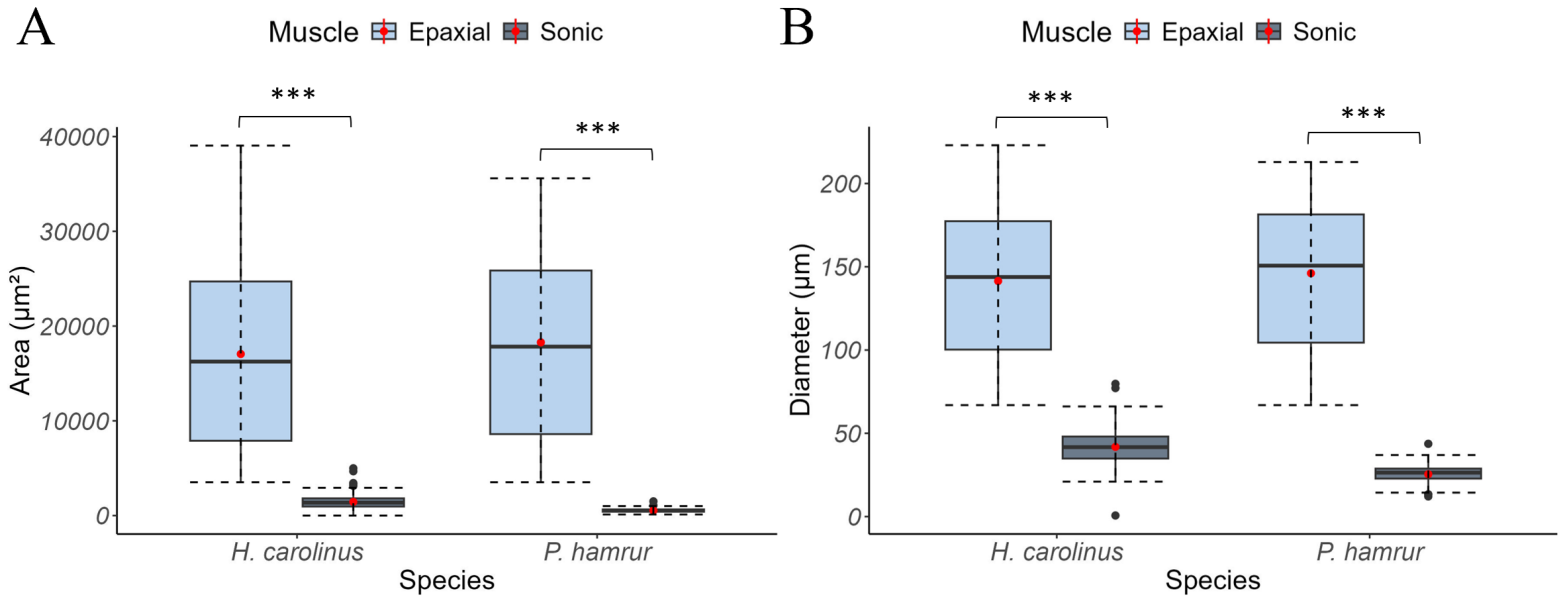
Boxplots representing the (A) area and the (B) diameter of sonic and epaxial muscles. The box boundaries represent the first and third quartiles, the whiskers indicate the minimum and maximum values, and the lines indicate the medians. The asterisks indicate how statistically significant the differences are between the epaxial and sonic muscles: ***P < 0.001).

In *H. carolinus*, sonic muscle fibres are 3.5 times smaller than epaxial fibres (Mann–Whitney U = 12, *p* < 0.0001; Fig. 7). Their mean diameter and cross-sectional area are 41 ± 10 µm (*n* = 277) and 1,488 ± 664 µm^2^ (*n* = 277), respectively, compared to 141 ± 41 µm (*n* = 109) and 17,954 ± 9,071 µm^2^ (*n* = 109) for the white epaxial fibres (Fig. 8). In *P. hamrur*, sonic fibres are 6 times smaller than epaxial fibres (U = 0, *p* < 0.0001). The mean diameter and area are 25 ± 5 µm (*n* = 138) and 534 ± 223 µm^2^ (*n* = 138), respectively, compared to 146 ± 44 µm (*n* = 47) and 18,282 ± 9,893 µm^2^ (*n* = 47) for the white epaxial fibres (Fig. 8). For reference, the diameter of sonic muscle fibres differs significantly between the two species (U = 2,152, *p* < 0.0001), while epaxial fibre diameters are not significantly different (U = 2,355, *p* = 0.486).

## Discussion

In this study, the comparison of sound-producing mechanisms in two species from different genera reveals that the ability to produce sound relies on a shared functional principle. While the presence of extrinsic sonic muscles attached to the anterior swim bladder region is a common feature arising through evolutionary convergence, the horizontal arrangement of muscle fibres originating from the ribs appears to be an original configuration. To our knowledge, a similar arrangement has only been reported in a few serrasalmid species (Mélotte et al., 2019). In both species, the relationship between pulse period and fundamental frequency clearly supports the forced response model, in which the contraction rate of the sound-producing muscles determines the vibration frequency of the swim bladder, thereby setting the fundamental frequency of the sound (Sprague, 2000; Fine et al., 2001; Rice & Bass, 2009; Millot, Vandewalle & Parmentier, 2011; Banse et al., 2021). This is further supported by the markedly smaller diameter of sonic muscle fibres, which is a well-known feature of this type of mechanism: in such systems, sonic fibres are consistently smaller than those of the epaxial musculature. In phylogenetically unrelated species, sonic fibres can be 2 to 21 times thinner than epaxial fibres (Mok et al., 2011; Kéver et al., 2014; Parmentier et al., 2014).

We also hypothesized that fish from geographically distant populations might produce acoustically distinct sounds. However, these differences are more strongly associated with size variation among specimens than with geographic origin. Indeed, in the PCA, specimens clustered according to size (juveniles *vs* adults) regardless of their region of origin. It should be noted, however, that only juveniles were available from the Pacific populations, whereas adults were only recorded from the Indian Ocean. Therefore, we cannot entirely exclude the possibility that acoustic differences may exist between adults from the two ocean basins. Despite the correction of the acoustical features for the size difference between individuals, this segregation between juveniles and adults remains. Interestingly, these differences are not limited to the acoustic features alone but also appear in the oscillogram profiles. In juveniles, pulses are produced in a continuous manner, typically consisting of a single peak per pulse. In adults, however, sounds appear to be structured as sequences of grouped peaks (Fig. 3), that are more spaced. Interestingly an analogous situation is found in *Halobatrachus didactylus* where juveniles mostly produce single grunts, whereas sexually mature individuals emit several trains of grunts that are however each shorter than in juveniles (Vasconcelos & Ladich, 2008). As in *H. carolinus*, vocalizations in *H. didactylus* show clear changes across developmental stages in terms of temporal structure, spectral content, and intensity. Given that the anatomy of the sound-producing apparatus remains similar between juveniles and adults in *H. carolinus*, the differences in acoustic features may be attributed either to the size of the sonic structures, to muscle anatomy or to neurophysiological differences within the sonic system.

Within the Priacanthidae family, sound-producing muscles have been identified in species of *Heteropriacanthus* and *Priacanthus*, but not in *Pristigenys* or *Cookelous*, which do not possess sound-producing muscles and are therefore considered mute (Shao & Chang, 1985; Starnes, 1988). *Heteropriacanthus* and *Priacanthus* differ in both swim bladder morphology and the configuration of their sonic muscles. In *Priacanthus*, the swim bladder has anterior projections associated with the saccular fossae of the skull, a feature that is absent in *Heteropriacanthus*. The insertion of sonic muscles also differs: in *Priacanthus*, they attach to the anterior portion of the swim bladder either ventrolaterally or ventromedially, whereas in *Heteropriacanthus*, the insertion is anteromedial.

It appears that, over the course of evolution, the shift of the sonic muscle from a frontal to a more ventral position in *Priacanthus* allowed for the development of these anterior projections. Interestingly, a comparable difference in swim bladder organization has also been observed within the Holocentridae family: members of the Holocentrinae subfamily lack cranial contact (but see *Holocentrus* species), while anterior swim bladder projections are present in the Myripristinae (Nelson, 1955; Parmentier et al., 2011; Banse et al., 2024). It has been experimentally shown in Myripristinae that the anterior projections are associated with increased hearing sensitivity (Coombs & Popper, 1979). The similarity of configuration reasonably suggests that, as in Holocentridae, the presence of anterior swim bladder projections in *Priacanthus* may enhance auditory capabilities compared to *Heteropriacanthus*. Interestingly, anterior swim bladder extensions are also found in non-phylogenetically related species, such as certain Cichlidae (Schulz-Mirbach, Metscher & Ladich, 2012) and Gerreidae (Parmentier, Mann & Mann, 2011). In both cases, this configuration was shown to enhance auditory capabilities. All these cases provide a clear example of evolutionary convergence and support enhance auditory abilities in *Priacanthus species*.

## Conclusions

In Priacanthidae, the evolutionary shift in sonic muscle position does not correspond to any significant change in sound structure, as the oscillograms and acoustic features of *H. carolinus* and *P. hamrur* are broadly similar. This supports the idea that the shift in sonic muscle position is primarily related to enhanced hearing abilities rather than to sound production itself. The comparison with Holocentridae extends beyond acoustic communication: both groups also share large eyes and predominantly reddish coloration—traits typically associated with a predominantly nocturnal lifestyle. Given their similar ecological niches, it is striking to observe how environmental pressures appear to have driven comparable evolutionary adaptations in both groups.

##  Supplemental Information

10.7717/peerj.20821/supp-1 Supplemental Information 1 Results of the regression analyses between size (TL) and acoustical variables of sounds in * H. carolinus.* * P* values in bold are < 0.05.

10.7717/peerj.20821/supp-2 Supplemental Information 2 Variable loadings on the first two principal components (PC1 and PC2)

## Additional Information and Declarations

### Data Availability

The following information was supplied regarding data availability: The data is available at Figshare: Banse, Marine (2026). Data_Sounds_Priacanthidae. figshare. Dataset. https://doi.org/10.6084/m9.figshare.29492237.v1
The data is available at MorphoSource: - https://doi.org/10.17602/M2/M765108
- https://doi.org/10.17602/M2/M765146.
