# Supplementary material for: Ontogenetic shifts in sound production and shared sonic mechanisms in two priacanthid fishes
Source: PeerJ. 2026 Feb 26;14:e20821. doi: 10.7717/peerj.20821 (PMC12950183; doi:10.7717/peerj.20821)
Supplement: Supplemental Information 1 — P values in bold are < 0.05. [file peerj-14-20821-s001.docx]

| Variable *X* | Formula | Slope estimate | Intercept estimate | Multiple R^2^ | *P* |
| --- | --- | --- | --- | --- | --- |
| Duration | *X* ~ TL | 0.42 | 216.65 | 0.00 | 0.903 |
| Number of pulses |  | -2.01 | 66.94 | 0.48 | **0.018** |
| F0 |  | -10.52 | 338.49 | 0.97 | **0.000** |
| Fpeak |  | -10.91 | 697.97 | 0.58 | **0.007** |
| Period 1 |  | 0.64 | 2.05 | 0.87 | **0.000** |
| Perm |  | 0.81 | -2.60 | 0.91 | **0.000** |
