# Supplementary material for: Ontogenetic shifts in sound production and shared sonic mechanisms in two priacanthid fishes
Source: PeerJ. 2026 Feb 26;14:e20821. doi: 10.7717/peerj.20821 (PMC12950183; doi:10.7717/peerj.20821)
Supplement: Supplemental Information 2 [file peerj-14-20821-s002.docx]

| Variable | PC1 | PC2 |
| --- | --- | --- |
| Sound duration | -0,28103254 | -0,98581819 |
| Fundamental frequency | -1,05247919 | 0,40142506 |
| Dominant frequency | -1,04008726 | 0,3763666 |
| Number of pulses | -1,03695845 | -0,16312869 |
| Period 1 | -0,44985587 | -0,5676945 |
| Middle pulse period mean | 0,96562886 | 0,11635967 |
